# F111. ELECTROPHYSIOLOGICAL CORRELATES OF AVOLITION-APATHY DOMAIN IN SCHIZOPHRENIA

**DOI:** 10.1093/schbul/sby017.642

**Published:** 2018-04-01

**Authors:** Giulia Giordano, Thomas Koenig, Armida Mucci, Annarita Vignapiano, Antonella Amodio, Giorgio Di Lorenzo, Cinzia Niolu, Mario Altamura, Antonello Bellomo, Silvana Galderisi

**Affiliations:** 1 University of Campania “Luigi Vanvitelli”; 2 Translational Research Center, University Hospital of Psychiatry Bern; 3 University of Rome Tor Vergata; 4 University of Foggia

## Abstract

**Background:**

Negative symptoms represent a core feature of schizophrenia. They have been associated to poor functional outcome, worse quality of life and poor response to pharmacological treatment. Several factor analytic studies have reported that negative symptoms can be divided into two domains referred to as Avolition-apathy which includes Avolition, Anhedonia and Asociality and the Expressive deficit domain, which includes Alogia and Blunted affect.

Avolition-apathy has been associated to a dysfunction of brain circuits involved in motivation, in particular to those related to the ability to anticipate pleasure and learn from rewards. It is highly controversial whether Avolition-apathy and all subcomponent symptoms share the same neurobiological underpinnings.

Our study, using brain electrical microstates (MS) which reflect global, subsecond patterns of functional connectivity, had two primary aims: 1) to identify differences between healthy controls (HC) and clinically stable people with schizophrenia (SCZ) in brain electrical microstate parameters and 2) to investigate the associations of the microstate parameters with the Avolition-apathy domain and its subcomponent symptoms.

**Methods:**

We analyzed multichannel resting EEGs in 142 SCZ and in 64 HC, recruited within the add-on EEG study of the Italian Network for Research on Psychoses. The microstate analysis was performed using an in-house plugin for Brain Vision Analyzer. Based on the microstate map templates from a large normative study, each moment of the ongoing EEGs was assigned to one of four microstates (MS) classes (MS-A, MS-B, MS-C, MS-D). Microstates were then quantified in terms of relative time contribution, duration and occurrence. Negative symptoms were assessed using the Brief Negative Symptoms Scale (BNSS): Avolition-apathy was obteined by summing the scores on the subscales Anhedonia (consummatory and anticipatory anhedonia), Avolition and Asociality; Expressive deficit was computed by summing the scores on the subscales Blunted Affect and Alogia.

Analysis of variance (ANOVA) was used to test group differences on MS parameters. Pearson’s r coefficients were computed to investigate the correlations of MS parameters with the negative symptom domains and subcomponent symptoms.

**Results:**

There was no significant group difference in sex (p=0.073) and age (p=0.547) between SCZ and HC. SCZ, in comparison to HC, showed increased contribution (p=0.009) and duration (p=0.016) of MS-C.

As regard to negative symptoms, the total score of the BNSS was positively correlated with the contribution of MS-A (r= 0.19, p<0.03). Avolition-apathy domain (r=0.22, p<0.01), anticipatory anhedonia (r=0.20, p=0.02), avolition (r=0.20, p=0.02) and asociality (r=0.25, p=0.003), but not consummatory anhedonia (r=0.13, p=0.13), were positively correlated with the contribution of MS-A. There was no correlation between Expressive deficit and MS-A parameters.

**Discussion:**

Our findings, in line with previous studies, showed an increased contribution of MS-C in SCZ. MS-C was not associated with clinical features, thus probably representing a trait marker of the disease. In addition, our results support different neurophysiological correlates of the two negative symptom domains and suggest that only anticipatory anhedonia, but not consummatory anhedonia, might be linked to the Avolition-apathy domain. These findings are in line with studies reporting an intact ability to experience in the moment pleasure and an impairment in pleasure anticipation (anticipatory anhedonia) in people with schizophrenia.

